# Optic Perineuritis Presenting with Transient Monocular Vision Loss (TMVL): Case Report

**DOI:** 10.2147/IMCRJ.S460611

**Published:** 2024-07-08

**Authors:** Mohamed M Tawengi, Mohamad Fael, Rizeq F Hourani, Tamader Alyaarabi, Abdelaziz M Tawengi, Gamal Alfitori

**Affiliations:** 1 College of Medicine, Qatar University, Doha, Qatar; 2 Department of Internal Medicine, Hamad General Hospital, Doha, Qatar

**Keywords:** optic perineuritis, optic neuritis, transient monocular vision loss

## Abstract

Optic perineuritis is an inflammatory condition that presents with reduced visual acuity and painful eye movement. The presentation of optic perineuritis is similar of optic neuritis which results in delayed diagnosis and management. Up to this date, we found a single case of optic neuritis that presented with transient monocular vision loss (TMVL). No cases of optic perineuritis were associated with TMVL. Here, we report a case of a 30-year-old woman who presented with recurrent attacks of painless vision loss in her left eye, reaching up to 30 attacks per day. Ophthalmological examination was otherwise unremarkable. Lab investigations were normal. Magnetic resonance imaging was done, which showed left optic nerve sheath enhancement suggestive of left-sided focal optic perineuritis. Patient was managed with 1 mg IV methylprednisolone for 3 days. We report this case to shed light on the importance of accurate and early diagnosis of optic perineuritis presenting with TMVL. Prompt management of optic perineuritis is crucial in reducing morbidity and risk of relapse.

## Introduction

Optic perineuritis, also known as perioptic neuritis, is an uncommon condition that is characterized by inflammation of the optic nerve sheath.^1^,^2^ OPN has been associated with autoimmune conditions but may also be idiopathic. OPN, in contrast to ON, is not linked to multiple sclerosis.^3^ Patients generally present with unilateral decrease in visual acuity with pain on eye movement.^2^,^3^

The mean age of presentation in several case series is between 40 and 60 years. A case study with 14 individuals with OPN reported a mean age of 41 years at presentation.^4^ Women are more likely to develop OPN.^1^

The clinical manifestations of OPN may resemble those of ON. Most patients first experience either subacute vision loss and/ or ocular discomfort. Like ON, eye movement may make eye pain worse, but OPN may cause eye discomfort that is more intense or persistent than ON. OPN can cause any degree of sight loss, from none, to severe. Patients may complain of “spots” in their vision, blurriness, fading, or splotches.^1^

OPN has never been reported to present with transient complete loss of vision, which was previously termed as amaurosis fugax. There has been one reported case of ON with transient monocular vision loss, however none with OPN.

This case report highlights a unique presentation of OPN in the form of recurrent TMVL, the term which usually implies a vascular cause for the acute transient visual loss. It will discuss the presentation, treatment, and prognosis of the patient. Finally, advice will be given on how to analyze and handle a patient who presents with OPN and TMVL.

## Case Report

### Background and Chief Complaint

Our patient is a 30-year-old Nepalese lady with unremarkable past medical and surgical history who presented to the hospital complaining of recurrent episodes of blindness in the left eye for one-week duration. She was referred from a Primary Health Care Center to Hamad General Hospital located in Doha, Qatar.

### History of Present Illness

According to our patient, she experienced attacks of blurred vision, followed by complete loss of vision in her left eye. She reported that blurring of vision lasted for few seconds followed by instantaneous loss of vision representing curtains falling over her vision. Each episode lasted 3–5 minutes, after which her vision recovers completely without blotching. She denied experiencing unusual fatigue, numbness, diplopia, slurred speech, or headache. She denied witnessing flashes of light, experiencing eye discomfort or photophobia. She also denied having eye pain, redness, or itching. She had no history of trauma, loss of consciousness, vomiting, fever, or weight loss. She stated that she experiences occasional, mild headaches which respond to simple analgesics.

The general physical examination was unrevealing. She was afebrile and was vitally stable A neurological examination confirmed that the patient was aware and attentive, with normal brain function. There were no localizing neurological impairments, and examination of the cranial nerves was unremarkable. She also underwent an ophthalmological assessment which was also unremarkable with 20/20 visual acuity, normal reactive pupils, no relative afferent pupillary defect, full color vision, and normal disc and macula. The patient was hospitalized for further workup of her transient monocular vision loss.

### Investigations and Imaging

Basic laboratory tests, including hemoglobin, red blood cells, platelets, and white blood cells with differentials, were normal on admission. The coagulation panel, as well as the renal, hepatic, and thyroid function tests were all unremarkable. Serum electrolytes, vitamin B12, lipid panel, glucose, CRP, and HbA1C% levels were all normal as well.

**Figure 1:**
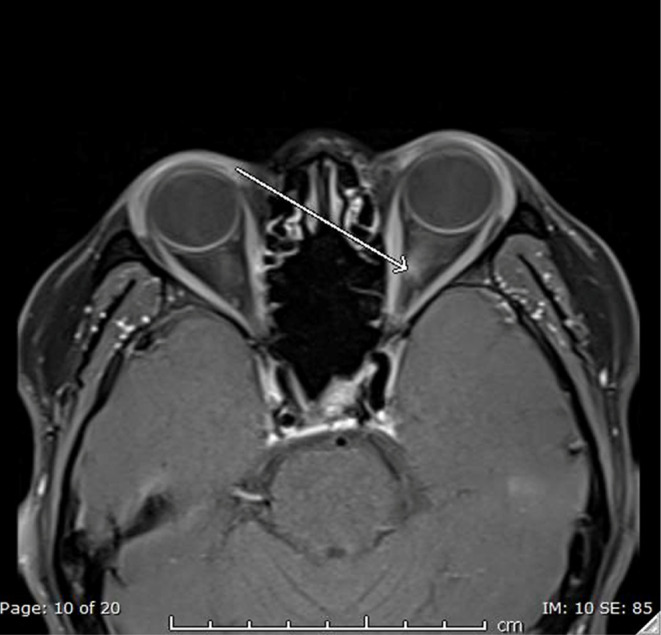
T1-Weighted Transverse Section with Fat Suppression. There is a signal of high intensity in the medial intracanalicular part of the left-sided optic nerve sheath (arrow).

**Figure 2:**
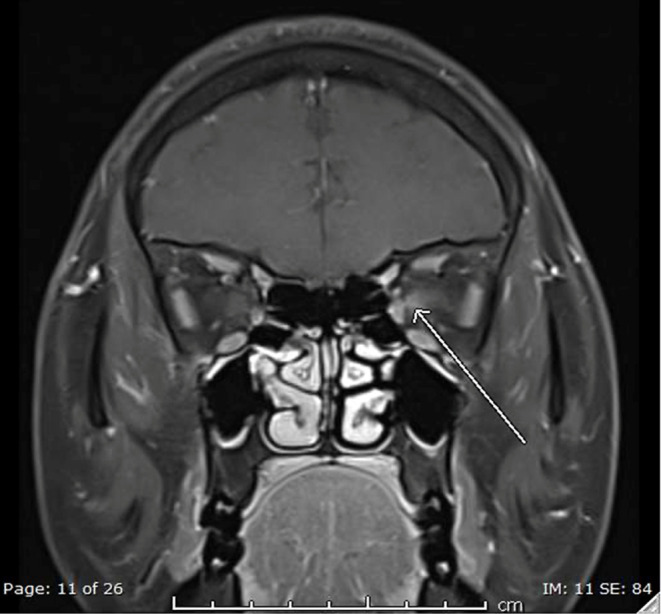
T1-Weighted Coronal Section MRI with Contrast. Contrast-enhancement in the medial intracanalicular part of the left-sided optic nerve sheath (arrow).

MRI orbit revealed a focal area of thickening and postcontrast enhancement in the medial aspect of the mid-intracanalicular part of the left optic nerve sheath for segment length of 8 mm (Figures 1 and 2, arrow) with subtle fat stranding in the same region. The right optic nerve appeared unremarkable. The rest of the intraorbital structures appeared unremarkable bilaterally. No localized brain parenchymal regions of diffusion limitation were seen on MRI. There were no signs of acute cerebral hemorrhage. The brain parenchymal signal intensity was grossly unremarkable. There was no midline shift or mass impact. The ventricular system and basal cisterns were still preserved. The structures of the posterior fossa were unremarkable. There was no abnormal brain parenchymal enhancement after contrast.

MRI of the spine showed normal spinal cord with no abnormal intermedullary T2 signal intensity or postcontrast enhancement. Neck and intracranial MRA appeared unremarkable with no major vascular occlusion, stenosis, or aneurysm formation.

The final impression was left optic nerve enhancement as described above, suggestive of focal optic perineuritis of the left optic nerve, which clinically correlated with the chief complaint of the patient.

Following the results of the MRI, further workup to discern the etiology of the findings was carried out. Autoimmune, atherosclerotic, or infectious etiologies were considered.

Patient was in normal sinus rhythm, with unremarkable contrast-enhanced echocardiography and carotid artery duplex ultrasonography. There were no suggestive features of tuberculosis or sarcoidosis on chest X-ray. Autoimmune panel including rheumatoid factor, anti-CCP, ANCA, C3, C4 was negative.

Additionally, a lumbar puncture was performed, and CSF examination revealed normal cell counts, protein levels, and glucose level. The level of IgG in the CSF was 44 mg/L (normal <34 mg/L) with a normal index of 0.5. There were no oligoclonal bands. Further analysis of CSF including VDRL screening, culture, cryptococcal antigen, and acid-fast bacilli smear and cytology was all negative. A viral panel tested negative for HSV1, HSV2, VZV, mumps, parecho virus, enterovirus, and COVID-19.

A visual evoked potential test was done and was normal for both eyes, with no evidence of optic neuropathy on either side.

### Treatment

The patient experienced repeated episodes of vision loss while in the hospital too, however, these episodes were shorter and lasted only for few seconds. She was started on 1 mg IV methylprednisolone for 3 days. She noted that her symptoms had improved after receiving treatment. Patient was discharged after 4 days with a follow-up appointment 3 weeks later.

### Follow-Up

At her follow-up appointment 3 weeks later, the patient disclosed that her symptoms had resolved completely and had not reoccurred. Physical examination was normal, and she was scheduled for a repeat MRI and a follow-up appointment in six months. Idiopathic left optic perineuritis was the final determined diagnosis.

## Discussion

OPN is a rare form of inflammatory orbital disease which has not been very well explored and most of the prior literature is in the form of case reports and case series. It is defined as an optic neuropathy where there is evidence of optic disc swelling but no evidence of optic nerve dysfunction, and the intracranial pressure is normal.^5^

OPN usually presents with painful eye movements, minor visual impairment, optic disc edema, and visual field abnormalities that include arcuate defects and peripheral island defects.^2^,^6^ However, this case was an unusual presentation of OPN where the patient presented with TMVL with atypically normal fundal examination and intact visual fields. This case aligns with previous studies indicating that optic perineuritis can present with atypical symptoms, making the diagnosis more challenging.

Certain autoimmune diseases have an established association with OPN including Graves’ disease, granulomatosis with polyangiitis, and IgG-4 disease which need to be ruled out.^7^ Findings on MRI including orbital fat inflammation, pachymeningitis, inflammation of optic sheath vessels, and optic nerve sheath edema are strongly suggestive of OPN.^7^ Nevertheless, our case highlights that such findings are not necessarily present, as our patient had an atypically normal fundal examination which is more frequently seen in ON. Therefore, the clinical, radiological, and laboratory investigations should be collated to make the diagnosis of OPN to avoid misdiagnosis with similar opthalmologic diseases including idiopathic demyelination optic neuritis.^7^

TMVL is defined as painless transient vision loss that lasts from seconds to minutes caused by lack of prefusion to the optic nerve and retina.^8^ The most common cause of TMVL is ischemia. However, it can also be neurogenic (eg, compressive optic neuropathies, papilledema, migraines, seizures) or idiopathic.^9^ Extensive investigations were conducted to evaluate the cause of TMVL in this patient including stroke workup, autoimmune panel, and screening for infectious causes. The only positive finding was left optic nerve sheath enhancement and focal thickening on MRI, which is a characteristic finding of OPN and provides distinction from ON.^3^,^4^,^10^ The pathogenesis and features of OPN are still lacking and it is still considered an idiopathic inflammatory disease when other inflammatory and infectious diseases are ruled out, like in our case.^11^

The unique presentation of this case and association with TMVL exemplifies the diagnostic difficulties. The challenge lies in identifying the condition early to provide appropriate management strategies. The timely and accurate diagnosis of OPN is crucial. In OPN, corticosteroid therapy results in rapid and dramatic improvement in vision and improves final vision outcome.^4^ Hence, prompt action to prevent irreversible vision loss from ischemic infarction using a high dose of intravenous steroids followed by a very slow tapering course of oral steroids as recurrence has been reported with a short duration of treatment.^3^,^4^

The unique presentation of this case with up to 30 attacks of painless vision loss per day, sets it apart from other documented instances. While previous literature has documented a single case of optic neuritis with TMVL, such association with optic perineuritis has not been reported. This observation raises questions about the underlying mechanisms and warrants further investigation into the potential connection between these two entities.

## Conclusion

Transient visual loss (TVL) reflects a heterogeneous group of disorders, some relatively benign and others with grave neurologic or ophthalmologic implications. Focused history and proper examination are the paramount to localize the problem and identify potential etiologies. The present case sheds light on the unique presentation of OPN as a transient sudden loss of vision. The diagnosis of OPN is challenging and requires prompt evaluation especially with such atypical presentation. Early diagnosis is crucial as steroid therapy is effective in improving symptoms and preventing long-term visual impairment.
